# Supplementary material for: Breakthrough SARS-CoV-2 Infections after Vaccination in North Carolina
Source: Vaccines (Basel). 2022 Nov 13;10(11):1922. doi: 10.3390/vaccines10111922 (PMC9695352; doi:10.3390/vaccines10111922)
Supplement: Supplementary file 1 [file vaccines-10-01922-s001.zip › vaccines-1982504-supplementary.pdf]

Table S1. Breakthrough analysis stratified by age.

Characteristics	Multivariate (Un-stratified)		Age: 18-44 (N=5,169)		Age: 45-64 (N=7,485)		Age: 65+ (N=3,104)	
Fixed covariates	**Hazard Ratio (95% CI)**	**P-value**	**Hazard Ratio (95% CI)**	**P-value**	**Hazard Ratio (95% CI)**	**P-value**	**Hazard Ratio (95% CI)**	**P-value**
**Sex**								
Female (*Reference group*)								
Male	1.11 (0.93 - 1.32)	0.2469	0.93 (0.68 - 1.26)	0.627	1.25 (0.98 - 1.6)	0.0718	1.09 (0.7 - 1.7)	0.7015
**Race/Ethnicity**								
Non-Hispanic White (*Reference group*)								
Non-Hispanic Black	0.69 (0.34 - 1.4)	0.303	0.4 (0.1 - 1.65)	0.2051	0.74 (0.28 - 1.95)	0.5376	1.95 (0.48 - 7.95)	0.3501
Hispanic	0.91 (0.4 - 2.07)	0.8148	1.23 (0.5 - 3.03)	0.6564	0.48 (0.07 - 3.33)	0.4567	---	---
Non-Hispanic Other	1.01 (0.59 - 1.73)	0.971	1.21 (0.59 - 2.51)	0.6021	0.63 (0.24 - 1.7)	0.3656	2.05 (0.56 - 7.48)	0.276
**Healthcare Worker Status**								
No (*Reference group*)								
Yes	1.12 (0.92 - 1.36)	0.2542	1.39 (1.04 - 1.85)	**0.0267**	0.96 (0.72 - 1.27)	0.766	1.89 (0.96 - 3.7)	0.0646
**Vaccination Brand**								
Pfizer (*Reference group*)								
Moderna	0.74 (0.6 - 0.91)	**0.0043**	0.85 (0.6 - 1.2)	0.3539	0.69 (0.51 - 0.93)	**0.0156**	0.74 (0.46 - 1.18)	0.208
Janssen (Johnson & Johnson)	1.29 (0.95 - 1.76)	0.1024	1.49 (0.92 - 2.41)	0.1038	1.19 (0.79 - 1.79)	0.4049	---	---
**Prior COVID Infection**								
No (*Reference group*)								
Yes	0.58 (0.39 - 0.85)	**0.0054**	0.5 (0.27 - 0.95)	**0.0348**	0.55 (0.32 - 0.95)	**0.0309**	1.15 (0.34 - 3.83)	0.8261
**County Classification**								
Urban (*Reference group*)								
Suburban	1.33 (1.07 - 1.63)	**0.0085**	1.52 (1.09 - 2.13)	**0.0147**	1.16 (0.85 - 1.57)	0.3503	1.72 (1 - 2.97)	**0.0498**
Rural	1.25 (1.01 - 1.53)	**0.037**	1.09 (0.76 - 1.56)	0.6424	1.38 (1.04 - 1.84)	**0.0255**	1.16 (0.67 - 2.02)	0.591
Time varying covariates								
**Vaccination Rate in County of Residence 3 weeks prior**								
≤ 60% (*Reference group*)								
> 60%	0.85 (0.64 - 1.13)	0.2582	0.84 (0.55 - 1.3)	0.4358	0.8 (0.52 - 1.25)	0.3303	1.23 (0.55 - 2.78)	0.6167
**Mask Usage in week prior**								
≤ 90% (*Reference group*)								
> 90%	0.66 (0.56 - 0.78)	**<0.0001**	0.52 (0.39 - 0.7)	**<0.0001**	0.79 (0.63 - 0.99)	**0.041**	0.65 (0.41 - 1.02)	0.0618
**Delta Time Frame ***								
Not in Delta Time Frame (*Reference group*)								
In Delta Time Frame	3.55 (2.35 - 5.36)	**<0.0001**	4.68 (2.47 - 8.89)	**<0.0001**	2.87 (1.61 - 5.12)	**0.0004**	3.66 (0.78 - 17.18)	0.0997
**Omicron Time Frame ****								
Not in Omicron Time Frame (*Reference group*)								
In Omicron Time Frame	16.63 (10.02 - 27.59)	**<0.0001**	27.42 (12.58 - 59.76)	**<0.0001**	12.43 (6.1 - 25.34)	**<0.0001**	5.27 (0.95 - 29.21)	0.057
**Has received booster shot**								

No (*Reference group)*								
Yes	0.33 (0.27 - 0.41)	**<0.0001**	0.33 (0.23 - 0.46)	**<0.0001**	0.33 (0.24 - 0.45)	**<0.0001**	0.27 (0.15 - 0.48)	**<0.0001**

* Delta Time Frame set as between June 28, 2021 and November 21, 2021, ** Omicron Time Frame set as between December 05, 2021 and January 03, 2022.
